# The interior environment design for entrepreneurship education under the virtual reality and artificial intelligence-based learning environment

**DOI:** 10.3389/fpsyg.2022.944060

**Published:** 2022-11-09

**Authors:** Wangting Li, Zhijing Xue, Jiayi Li, Hongkai Wang

**Affiliations:** ^1^Academy of Arts, Shandong University of Science and Technology, Qingdao, China; ^2^College of Art and Design, Zhengzhou University of Industry Technology, Zhengzhou, China; ^3^College of Business, Gachon University, Seongnam, South Korea; ^4^Academy of Arts and Design, Tsinghua University, Beijing, China; ^5^College of Journalism and Communications, Shih Hsin University, Taipei, China

**Keywords:** artificial intelligence, entrepreneurship education, interior environment design, virtual reality technology, 3D modeling

## Abstract

Nowadays, with the rapid growth of artificial intelligence (AI), entrepreneurship education has attracted more and more attention from society. To this end, it is necessary to gradually transform the traditional teaching mode into a new type of teaching that is more innovative, practical, and inclusive and in line with entrepreneurship education. The focus of the teaching mode change is on the optimization of the teaching environment. For this purpose, a method derived from distributed virtual reality (DVR) technology is specially designed. It refers to the fact that multiple users can join together through a computer network and participate in a virtual space at the same time to experience the virtual experience together. Based on this, the distributed 3D interior design is innovatively proposed. The innovation is mainly reflected in the application of VR technology, which is different from traditional software design. According to the functions and needs of the entrepreneurship teaching environment, first, the distributed feature information is collected, and second, the corresponding color image model is constructed by the fusion method, and edge contour detection and corresponding feature data extraction are carried out for the distributed image. Using a Red, Green, and Blue (RGB) color decomposition method, the pixel feature decomposition of spatially distributed image color is performed. And the feature reorganization of the 3D point cloud is combined to optimize the color space and color features of the combined design. On this basis, the distributed 3D interior design system is designed with VR and visual simulation technology. Finally, the Three-Dimensional Studio Max (3ds MAX) is used to establish 3D modeling, and the modeling software Multigen Creator is adopted to carry out the hierarchical structural design. The test results manifest that the Normalized Root Mean Square Error (RMSE) and information saturation of the distributed 3D interior design are reduced by 0.2 compared with the traditional design, the time overhead is shortened to one-sixth of the original, and the effect is more in line with the design requirements. It is hoped that this design method can provide new ideas and new perspectives for the optimization of the entrepreneurship teaching environment.

## Introduction

It is demonstrated from the data released every year that entrepreneurship has become a new source of power for China's economic development. Because colleges and universities are the basic stations for cultivating talents, they are very critical in China's innovation and entrepreneurship system. The focus of entrepreneurship education is on cultivating students' international vision and strengthening the connection between classroom content and the actual social situation through the combination of theory and practice. The core is an innovative development model guided by the needs of social occupations (Hameed et al., 2021). It is well known that the success of entrepreneurship education depends on whether students have the correct entrepreneurial values and professional technical support. Therefore, the inconsistency between domestic entrepreneurial individuals and the social nature of entrepreneurship education has led to the unreasonable layout of entrepreneurship practice bases in colleges and universities. The function is not accurate enough, which ultimately makes it impossible for students to have a good experimental environment at school, and there is a general problem of engaging in idle theorizing. From this, it is analyzed that three conditions must be met to achieve the goal of entrepreneurship education in colleges and universities: First, the requirements of the ecological system of colleges and universities must be met. Entrepreneurship education is based on “colleges and universities”. Hence, it is necessary to consider various factors such as the organization and management of colleges and universities, curriculum design, and teacher resources. Second, the entrepreneurship education model in colleges and universities must first find the right criteria, not only to meet the actual needs of society, but also to take care of the actual educational situation, both of which are indispensable. At last, due to the different professional advantages and their own historical factors of each university, the model should be adapted to local conditions and eclectic, which means that different models should be developed by combining the core of entrepreneurship education and the school's own level, type, and regional characteristics (Jardim et al., 2021).

Now, in architectural design, the common methods of computer vision feature extraction are mainly scale-invariant feature transformation (SIFT), histogram of oriented gradient (HOG), etc. SIFT mainly simulates the multi-scale features of image data. The large-scale grasps the general features, and the small-scale emphasizes the details. The advantage is that the local features of the obtained image remain invariant to rotation, scale scaling, and brightness changes, while also maintaining a certain degree of stability to viewing angle changes, affine transformations, and noise. However, the real-time performance is not high, the feature points are few, and the features cannot be accurately extracted for objects with smooth edges (Hameed et al., 2021). HOG constitutes features by calculating and counting local areas of the image. Due to the small cell size of its features, a certain spatial resolution can be preserved. And the normalization operation makes this feature insensitive to local contrast changes. SIFT has good properties for feature extraction of objects in complex environments, while HOG has good properties for feature extraction of rigid objects. The image contour detection currently mainly uses the OpenCV contour detection function, which in turn draws the contour by reading the grayscale image of the object, binarization, and contour detection. Due to the different operators used, there will be different effects (Jardim et al., 2021). In the research, the methods of feature extraction, color-space development, fusion, and contour detection will be used concretely. At present, more research about entrepreneurship education are being carried out both domestically and abroad on the expansion of its model and content. However, there is no design method or interior environment design that incorporates entrepreneurship education. For example, Talmage studied common themes in dark literature, and provided the integration of dark-light language into entrepreneurship education (Talmage and Gassert, 2020). Verduijn proposed a pedagogical approach to entrepreneurship education based on an iterative and interactive process that oscillates between deconstructing and reconstructing entrepreneurship to create space for invention in the classroom (Verduijn and Berglund, 2020). Irawanto explored new generation of students' perceptions of higher entrepreneurship education by observing whether it has an impact on innovative behavior (Irawanto and Novianti, 2021). The teaching space of entrepreneurship education, which is not valued by researchers but is equally vital, started late. It is denoted that most domestic colleges and universities only place it on entrepreneurship guidance courses, and more are only in form without content. Moreover, there is no perfect teaching environment, which means there is an urgent need for the optimization of the teaching environment of entrepreneurship education (Chen et al., 2021).

For the interior environment design of entrepreneurship education, first, the advantages of Virtual Reality (VR) technology are innovatively used to design a new distributed 3D interior design system. The real space environment experience brought by VR technology is fully utilized, allowing students and teachers to participate in the design process more freely. Second, the optimization of spatial color and structure is processed and the color features are decomposed, and the visual collocation of different styles of the interior is performed. Finally, a distributed 3D visual reconstruction method is used to develop the design. Meanwhile, Artificial Intelligence (AI) technology is adopted to realize the design and interaction of VR technology in the simulation environment. In the process, various 3D simulation software is used: MAYA, 3ds MAX, Java, VRML, Soft Image, Light Wave 3D, etc., to perfect the optimal design of this system and draw conclusions through experimental comparison and analysis.

## Methods

### Functions and requirements of the 3D interior design system

VR technology has virtuality that is close to or even beyond reality. With the development of multimedia technology, 3D graphics generation technology, multi-sensing interaction technology, and high-resolution display technology can be used to generate a 3D realistic virtual environment. Users can enter the virtual environment immersively through special interactive devices. AI is a discipline that studies the use of computers to simulate certain thinking processes and intelligent behaviors of humans (such as learning, reasoning, thinking, planning). It mainly includes the principle of computer realization of intelligence, the programming of computers similar to human brain intelligence, so that computers can achieve higher-level applications. In recent years, with the progress of AI, its technology has been applied to motion trackers, force feedback devices, speech recognition, and other equipment, which has greatly improved the experience of VR technology. It can be said that in the AI environment, VR technology can be practical in many fields including architectural design.

Entrepreneurship education in universities is mainly to educate students to develop personal entrepreneurial thinking, enable them to realize their own entrepreneurial behaviors (Bian et al., 2021), and to draw conclusions on entrepreneurship education combined with the conclusions of the study of Borges et al. (2021). According to the above knowledge and concepts, a distributed 3D design system is created for the indoor environment of entrepreneurship education through the design technology of VR. It is well known that VR technology integrates AI, computer, graphics, etc. The purpose is to be immersive for users, so it is necessary to provide real images and other factors to simulate the virtual environment, so that users can feel that they enter the real physical environment. It is also possible to interact with the simulated 3D environment in real-time as in reality. Therefore, there is a need to create a 3D simulated environment through interactive software and hardware to achieve the goal (Abich et al., 2021). In terms of design and utilization, works can be presented in the form of a 3D virtual world, which can not only express the concept of creative design in an overall way, but also change the way and concept of the design (Fukuda et al., 2021).

For the interior environment design of entrepreneurship education, VR technology should be used to meet the requirements: (1) The distributed 3D interior design system of VR technology can transform the real interior environment into a visual virtual simulation. And the soft interior design environment requires bright but not dazzling lights and necessary color matching can make full use of the temperature of colors and the convergence of lights to create a classroom atmosphere. (2) The design system mainly includes structures, classroom appliances, teaching equipment, and other models. To design the model library through the above-required models, it is required that the designer can directly and easily integrate the required models into the design through the input model library. (3) The data previously combined with the virtual scenario model should be modified according to the needs of students and teachers in entrepreneurship education, so that the final design effect can be checked and adjusted at any time, to determine the most suitable plan for entrepreneurship education (Moussa et al., 2022). The above interaction process can be completed through the common Java, VRML communication principle. To realize real-time dynamic control of the scene in VRML, the Java program must access the scene with authority to realize the reception and transmission of information, and obtain scene objects through requirements (Sorin, 2021).

### Design of the interior model

**Figure 1:**
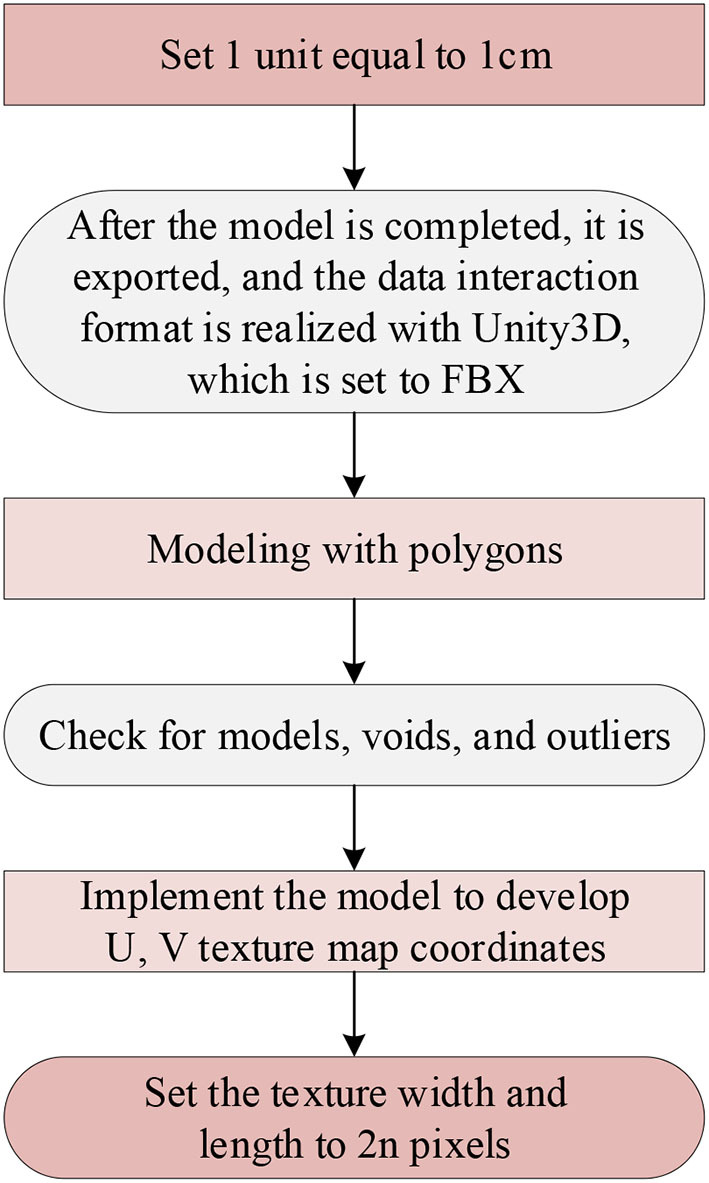
Technical details required to create a model.

The interior model of entrepreneurship education mainly comprises models of structures, classroom appliances, teaching equipment, and slogans. 3dsMAX is used as a modeling tool, and the details of its technical requirements are exhibited in Figure 1.

In Figure 1: (1) First set in 3dsMAX: 1 unit is set to 1cm, but it is displayed as m, because the 1:1 scale is used to complete the design of the model, and the ultimate result is to ensure that users browse with normal visual proportions in the VR device, increasing the sense of immersion. After completion, the file is exported to interact with the Unity3D data format. It should be set to filmbox (FBX), and the unit is still cm. It aims to make the ratio of FBX after importing the model in Unity3D to be 1:1. (2) Use polygons for modeling although not too many, otherwise, it will reduce the real-time rendering speed of the program. After the model is completed, check the polygons, and check whether the normal direction of the model is appropriate. Then the virtual solder joints, isolated points, etc. of the image are detected, and finally, the historical records of the process are sorted out (Bao, 2021). (3) To display the model texture reasonably, it is necessary to realize the texture production model through the UV texture map coordinates. There are two common types of maps: one is the diffuse reflection map: the purpose is to display the surface reflection and color of the object. The other is the bump map: it can enhance the three-dimensional sense of the model without changing the shape of the model. The rest of the normal map can change the slope of the model surface. The height map can change the height of the model surface, which can fully display the characteristics of the model surface through different lighting conditions. The Ambient Occlusion (AO) map can change the distance between each face of the model, so that different ambient occlusion maps can be generated. The conclusion is that various maps can be changed and viewed, at will, due to the needs of use (Hegazy et al., 2021). (4) The width and length of the texture should be set to 2n pixels for later application of Unity3D (Gan et al., 2021).

**Figure 2:**
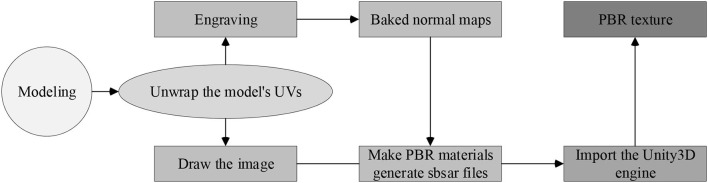
Workflow for processing into PBR materials.

To be more realistic, it is necessary to import data through Unity3D and carry out a series of physical dynamic rendering to complete the processing of various materials and lighting effects in the model, and to perform shading and Physically Based Rendering (PBR) through physical rendering. Nowadays, lighting processing is mainly realized through the PBR algorithm (Kim et al., 2021), which is a very realistic physical lighting model rendering algorithm. The physical parameters are directly adjusted to simulate realistic light, and the generated PBR textures are directly passed to the Shader to improve the scope of application and design efficiency of the textures (Apollonio et al., 2021), as shown in Figure 2.

In Figure 2, the model is first processed into a low-quality model in 3ds MAX, and then the model's UV is unfolded and processed into a higher-quality model through ZBrush. Next, the two quality models and textures are combined with PBR materials through Substance Painter software to generate sbsar data, and finally transferred to Unity3D to get the required PBR textures (Ryan, 2019).

### Collection and analysis of visual information points of design images

(1) Image sampling is performed first. To optimize the visual communication information, the Snake algorithm is used to decompose the edge contour features of the model's visual image in the design, and then adaptive information fusion enhancement is performed through the requirements. The Jacobian matrix *J*(*x, y*, σ) is expressed as (Tao et al., 2021):


(1) $$J\left(x,y,\sigma \right)=\left[\begin{array}{c} \frac{\partial P}{\partial x}\\ \frac{\partial P}{\partial y} \end{array}\right]=\left[\begin{array}{ccc} 1\& & 0\& & L_{X}(x,y,\sigma )\\ 0\& & 1\& & L_{Y}(x,y,\sigma ) \end{array}\right]$$

In equation (1), p represents the spatial point, *x* and *y* refer to the pixel position, and σ signifies the scale-space factor, and the size of the value indicates the smoothness of the image. Further, $\frac{\partial P}{\partial x}$ and $\frac{\partial P}{\partial y}$ express the first derivatives of the spatial point p with respect to *x* and *y*. L (*x, y*, σ) = *G* (*x, y*, σ) × *I* (*x, y*), where the *G*(*x, y*, σ) of the image is used as the seed point of the image, and the I (x, y) means the grayscale feature during fusion. The corresponding matching matrix *M* of the template can be obtained by histogram decomposition:


(2) $$M=\left[\begin{array}{cc} \frac{\partial^{2}P}{\partial x\partial x}\begin{array}{c} \to \\ N \end{array}\& & \frac{\partial^{2}P}{\partial x\partial y}\begin{array}{c} \to \\ N \end{array}\\ \frac{\partial^{2}P}{\partial x\partial y}\begin{array}{c} \to \\ N \end{array}\& & \frac{\partial^{2}P}{\partial y\partial y}\begin{array}{c} \to \\ N \end{array} \end{array}\right]=\left[\begin{array}{cc} L_{xx}(x,y,\sigma )\& & L_{xy}(x,y,\sigma )\\ L_{xy}(x,y,\sigma )\& & L_{yy}(x,y,\sigma ) \end{array}\right]$$

In equation (2), $\frac{\partial^{2}P}{\partial x\partial x}$, $\frac{\partial^{2}P}{\partial x\partial y}$, $\frac{\partial^{2}P}{\partial x\partial y}$and $\frac{\partial^{2}P}{\partial y\partial y}$ indicate the second derivative of the space point p to x and y, and $\vec{N}$ is the reference vector. The continuous 3D reconstruction method is used to realize the contour detection of the design image and the corresponding feature extraction.

(2) Reconstruction of visual features. To complete the processing of the 3D contour and feature data, the Linde, Buzo, and Gray (LBG) vector quantization method of the point-to-line model is adopted to mark the maximum gray value. The final image fusion center is denoted as d (*x, y*) (Banga et al., 2018). The gray pheromone is effectively extracted by designing the vector quantized feature quantity in the image, and the first k-dimensional image feature template is obtained, as shown in equation (3):


(3) $$\begin{array}{l} P\left(\Phi \right)=\int \frac{1}{2}{\left(\left|\nabla \Phi -1\right|\right)}^{2}dx \end{array}$$

In equation (3), Φ shows the feature quantity, and ∇Φ stands for the gradient of Φ. The matching items corresponding to the local template are represented by E^LBF^; the sampling components corresponding to edge pixels are represented by E_RGB_, and the fusion template of the visual image region can be obtained by sparse linear segmentation (Zarbakhsh and Demirel, 2018). The function *Data* of the fusion template is expressed as:


(4) $$\begin{array}{l} Data\left(x,y,d\left(x,y\right)\right)=|u\left(x\left(x,y\right),y\right)-u\left(x,y\right){|}^{2} \end{array}$$

In equation (4), it is specified that u and u are the reference template and the image to be reconstructed, respectively, |*u*(*x*(*x, y*), *y*)−*u*(*x, y*)|^2^ denotes the square of the corresponding difference vector between *u*(*x*(*x, y*), *y*) and *u*(*x, y*). Through the above steps, the designed 3D visual features (Li X. et al., 2020; Li C. et al., 2020) are reconstructed, and the processing process of the image is completed.

### Collision detection algorithm

**Figure 3:**
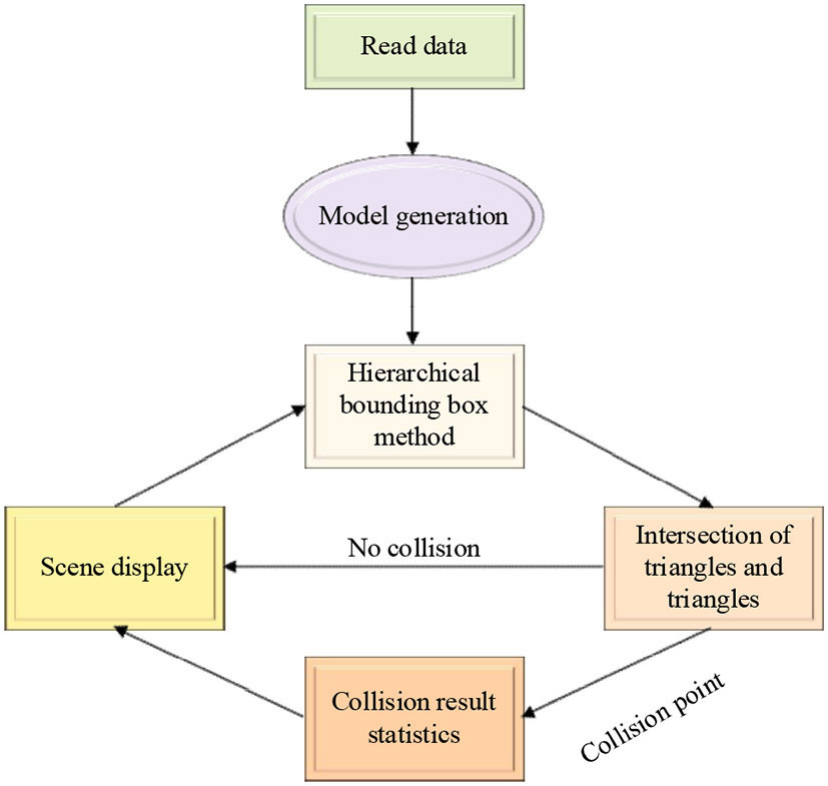
The system framework of the collision detection algorithm.

When using VR technology for Human-Computer Interaction (HCI), to make users feel a deep sense of immersion, it is necessary to deal with the collision of 3D scenes. The effectiveness of the bounding box-based method in collision detection is adopted to greatly speed up the collision detection algorithm. Its framework mainly includes the following modules: data reading, model generation, hierarchical bounding box, triangle and its intersection, collision result statistics, and scene display. The specific system framework of the collision detection algorithm is presented in Figure 3.

Figure 3 portrays that the data is read at the beginning of the system, and then a new model is generated in the system, and a hierarchical bounding box tree structure is established for the model and environment objects. Next, through the bottom-up update method, the position information of each particle in the object is obtained. In the subsequent accurate detection method, the triangle intersection module accurately calculates the specific collision point according to the basic geometric element information that may collide and transmits it to the collision result statistics module. Finally, the authenticity of the simulation is ensured by changing the coordinates of the collision particle position obtained by the collision result statistics module.

## Research methodology and system framework

### System development and design

**Figure 4:**
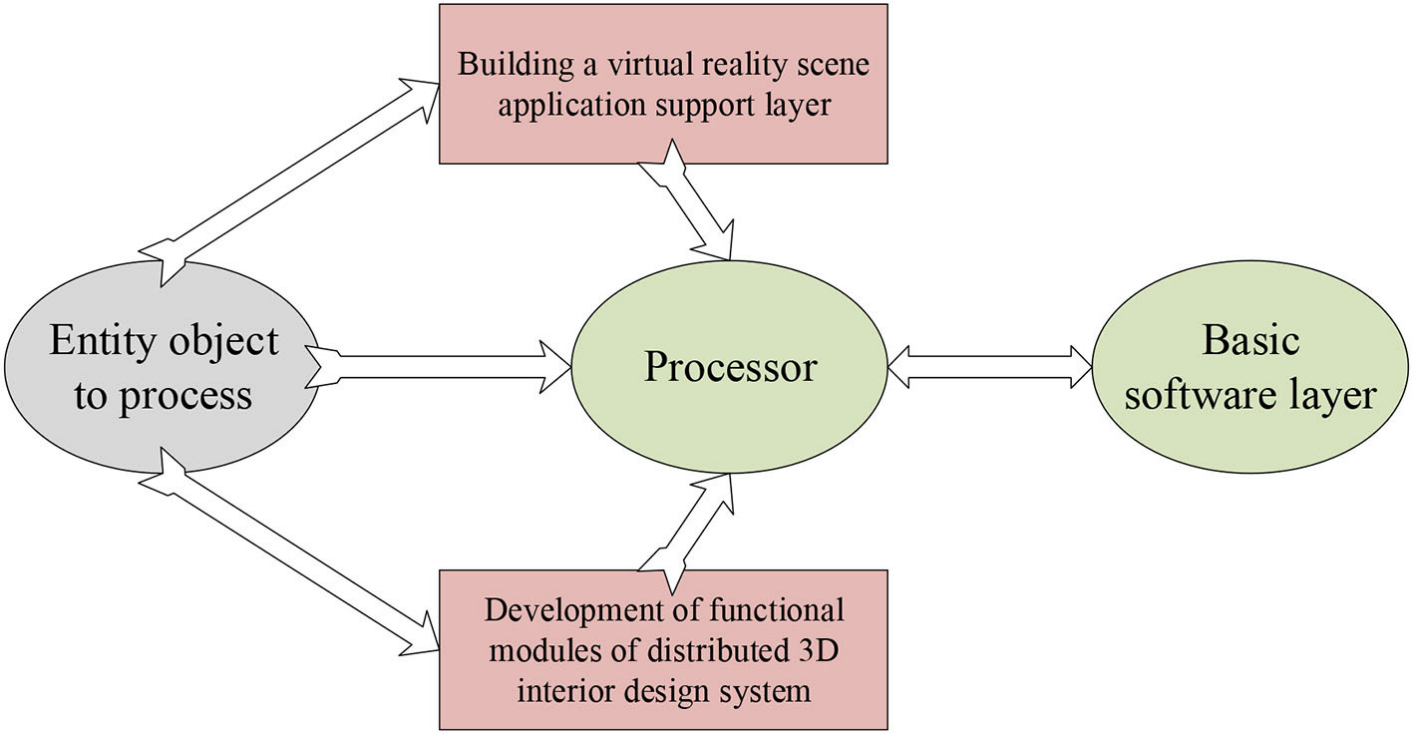
Diagram of the overall architecture design of the system.

After the image processing is completed, the design system should be developed. Through VR technology and visual simulation technology, the 3dsMAX software is used to realize the 3D modeling of the design, which is first processed into a hierarchical structure through the Multigen Creator software (Butcher et al., 2020). Then, the information transmission model is implemented through the Physical Cell Identifier (PCI) bus technology. The design system is regarded as a basic entity object, and the local information of the original design is processed through the multi-thread scheduling method. Next, the application support layer of the VR scene is built by using the client and server models, and the distributed 3D interior design system is researched and developed on the CCS 2.20 development platform. Finally, the integrated software layer is designed through the WEB-APP browser and the WEB-CULL server, and the network middleware is used to design the access service of the system and configure the middleware to complete various configurations of the system (Vogel et al., 2021). The details of the final structure are exhibited in Figure 4.

**Figure 5:**
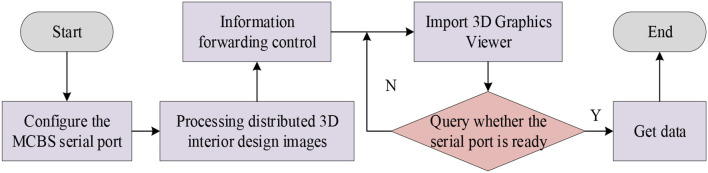
Development and realization process of distributed 3D interior design system.

The bus and module development and design of the system are carried out through the architecture design in Figure 4. The surface-level rendering method is used to realize the available real-time application files, and then according to the API dynamics in Vega Prime, change the program loading ways in the design. The control of program loading is realized through the local bus of PCI9054, and the signal is converted accordingly to complete the communication protocol development and bus scheduling of the design system. The Parallel Peripheral Interface (PPI) is used for human-machine communication, the embedded scheduling of the system is performed in the ARM Cortex-M3 core, and then compilation control and corresponding program reading and writing are performed through the design method in the form of Creator, and finally a hierarchical compilation platform is built (Milano et al., 2021). To improve the information receiving, forwarding, and processing skills of the system, it is also necessary to control the bus transmission at the application layer of the system, and grasp the realization of adaptive forwarding and appropriate link loading in the Simulink modeling software (Vishwakarma and Bhuyan, 2018). Eventually, the development process of the distributed 3D interior design system is obtained, as demonstrated in Figure 5.

Figure 5 manifests that in the modeling environment of Open Flight, the processed design system can be applied in VR, and the design effect can also be observed through the 3D graphics viewer in the compilation software (Jang et al., 2021).

### Design of human-scenario interaction

As the most challenging and comprehensive technology of AI, HCI covers various aspects such as semantic understanding, knowledge representation, language generation, logic, and reasoning. On the one hand, excellent HCI can bring intelligence to virtual objects and interactions; on the other hand, it also makes the development and production of VR content more intelligent and automated. VR can not only provide users with multiple scenarios for different entrepreneurial courses to choose from but also enable students and teachers to interact with the scenarios. It indicates that alternative scenarios need to be added to the design. For example, the room can be closed freely, and the user can open the door and walk into the room to observe; it is also possible to change the pattern in the wall and decide which light to use according to their own preferences so that the user can fully feel the specific situation before and after the change in the room. This requires designers to further intelligently design the interaction system, and deep learning under AI can enhance the intelligence of VR scenes.

**Figure 6:**
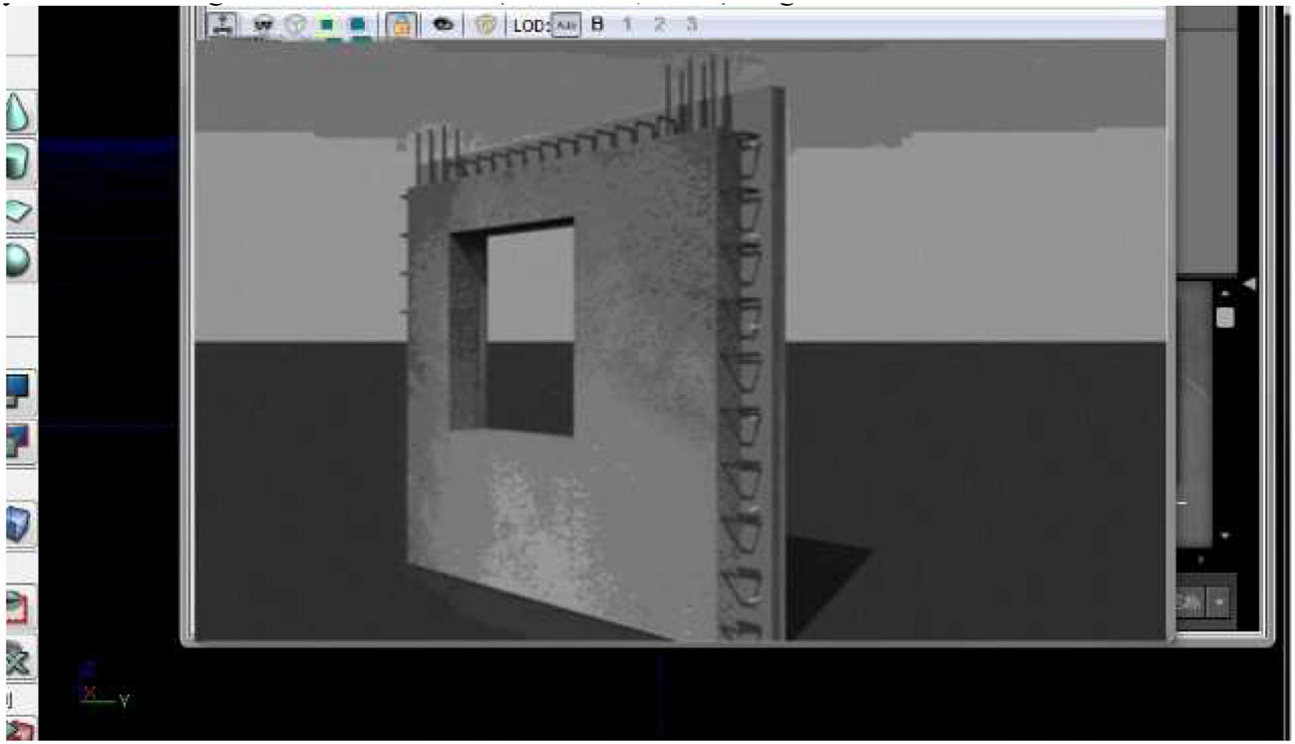
Demonstration of the 3D wall in the software. Reproduced with permission from CITN Lab Work Department, available at www.citn.ac.cn.

To fully satisfy the user's immersion in feeling, there is a need to deal with the collision of the 3D scene. After importing the model into the UDK (Unreal Development Kit), automatic positioning is realized at the origin of 3dsMAX. Coordinate settings are required in UDK to achieve fast position alignment. If the collision design is not performed in the 3D image during the detection process, automatic collision must be performed in UDK. There are two parts in the user's roaming process: path setting and animation processing. The first one is done using UDK cameras, in which the action trigger components are divided into two types of triggers, automatic and interactive, and interactive functions are performed through event triggers (Mилетић, 2020) . Compared with traditional design software, 3D virtual design is more convenient and easier for users to understand when to create the initial framework. For example, if a wall point is given, users only need to operate the software to input the corresponding data. Because of the continuous input, the two walls can be quickly connected between the current and newly inputted walls. It is denoted that in the process of creating the original architectural form, it can be quickly created through dimensional data (Han et al., 2019). Figure 6 shows the results of the 3D wall model.

**Figure 7:**
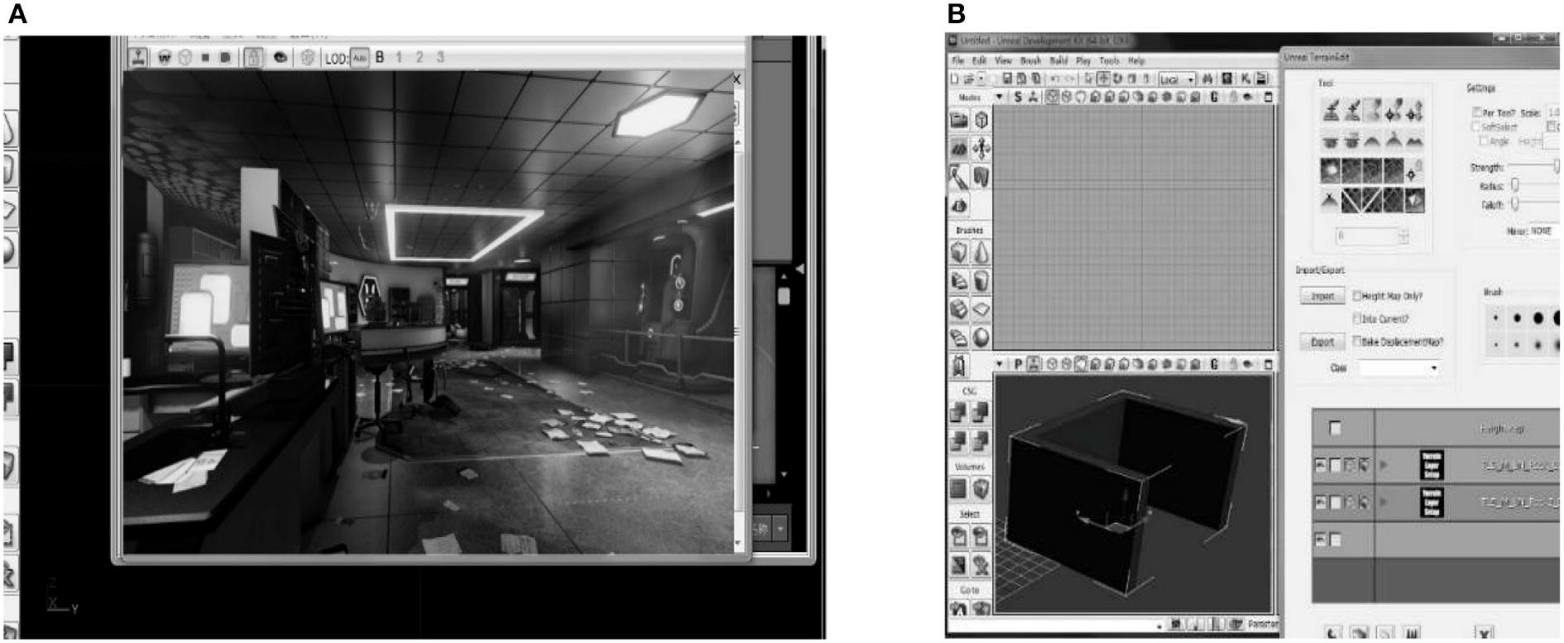
Schematic diagram of the application of the component modeling system [**(A)** is the assembly modeling software; **(B)** is the import process of the software model]. Reproduced with permission from CITN Lab Work Department, available at www.citn.ac.cn

Based on the designed room structure, only by modifying the data in the panel through the component modeling system, a complete set of doors and windows can be quickly created in the space reserved for doors, windows, and walls. After the replacement structure is selected, make personalized changes to its data. A schematic diagram of the application of the component modeling system is indicated in Figure 7.

Figure 7A signifies that the common structure can be changed quickly, and only simple operations through the mouse and keyboard are required. Therefore, it is necessary to use the parameter information of various objects that have been added as much as possible to shorten the post-processing time. The software system can import a large number of other decoration files through 3ds format and Mesh format and can realize functions such as zooming and moving. In the design of entrepreneurship education, the placement of desks, chairs, teaching aids and other such models are closely related to other structures in the design (Lazor et al., 2019).

The example in Figure 7B manifests that, through this function, different decorations and models can be freely changed, and the model can be modified to have a more suitable style and texture with the overall layout. It can also share resources with the team, share various existing design models and materials, and save the cost of different designs. In the design of environmental vision, the main task is to adjust the material, color, position, etc. of the scene objects according to different requirements. The most commonly used 3D design software is the Graphics Processing Unit (GPU) real-time rendering mode. Designers can simply modify the data for each object in the design to change the design effect. It allows users not only to see the effect after modification, but also to choose a design style by comparing different effects (Jing et al., 2018). Its main advantage is to enhance the client's authority in the design process, so the client will be satisfied with the final design result. Meanwhile, it also allows designers to deal with various details in advance according to different design performances, thereby reducing defects in the design process.

## Experimental results and discussion

### Research subjects and background

The distributed 3D interior design system is developed on the CCS 2.20 development platform. The integrated software layer is designed through the WEB-APP browser and the WEB-CULLserver, and the network middleware is used to design the access service of the system and configure the middleware to complete various configurations of the system.

### Setup of experimental data

The total number of sample sets of the designed images is set at 2000, all of which are obtained from the actual indoor environment of entrepreneurship education in a university. The size of the designed 3D visual block area is set to 550 × 550 × 220, which includes data such as walls, windows, front and rear doors, chandeliers, tables, and chairs. Due to space limitations, the specific data will not be presented here. The characteristic matching coefficient is set to 0.15 after the experimental comparison and analysis is the most suitable, and then the 3D interior design is realized through the visual simulation program.

### Test results of 3D interior design schematics

**Figure 8:**
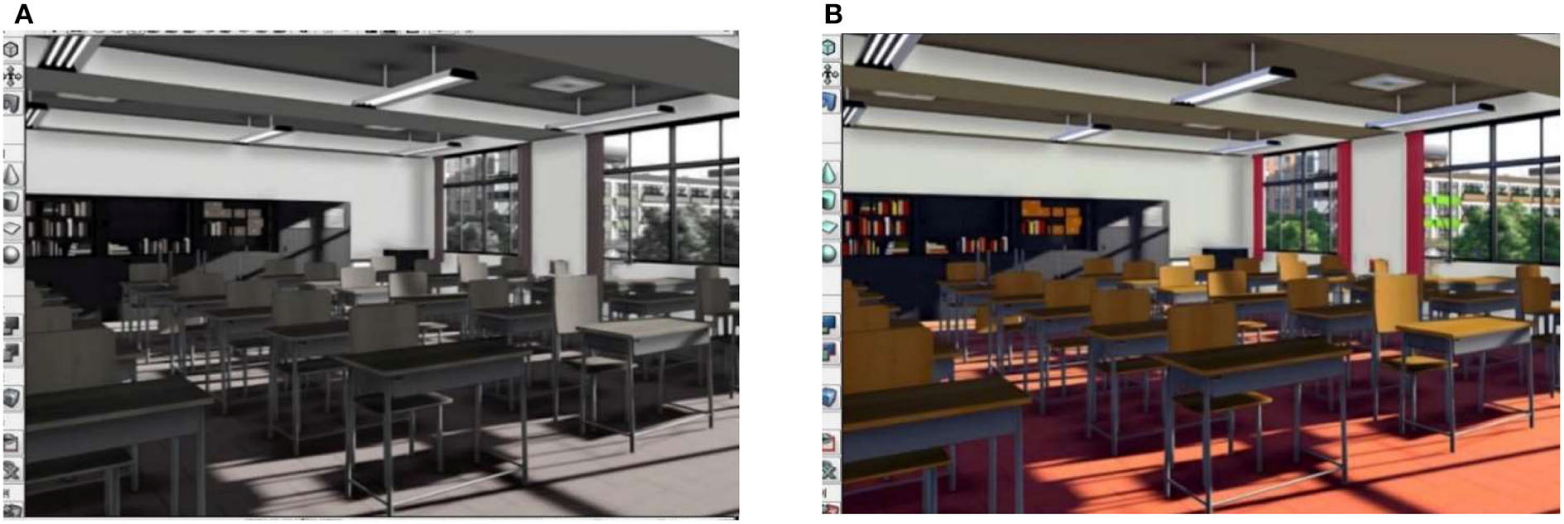
Schematic diagram of distributed 3D interior design [**(A)** is the rendering of 3D interior design, and **(B)** is the result of image information fusion]. Reproduced with permission from CITN Lab Work Department, available at www.citn.ac.cn

The schematic diagram of the design of the indoor environment for entrepreneurship education is illustrated in Figure 8.

In Figure 8A, the designed renderings basically fulfill the design requirements. Furthermore, by reconstructing the color space of the design image and fusing the feature reorganization of the 3D point cloud, the combination optimization of the realistic color feature of the interior design is obtained. The final design result is demonstrated in Figure 8B, and the color, light and shadow after the fusion of image information are basically in line with expectations.

### Experimental results and analysis of collision detection algorithm

**Figure 9:**
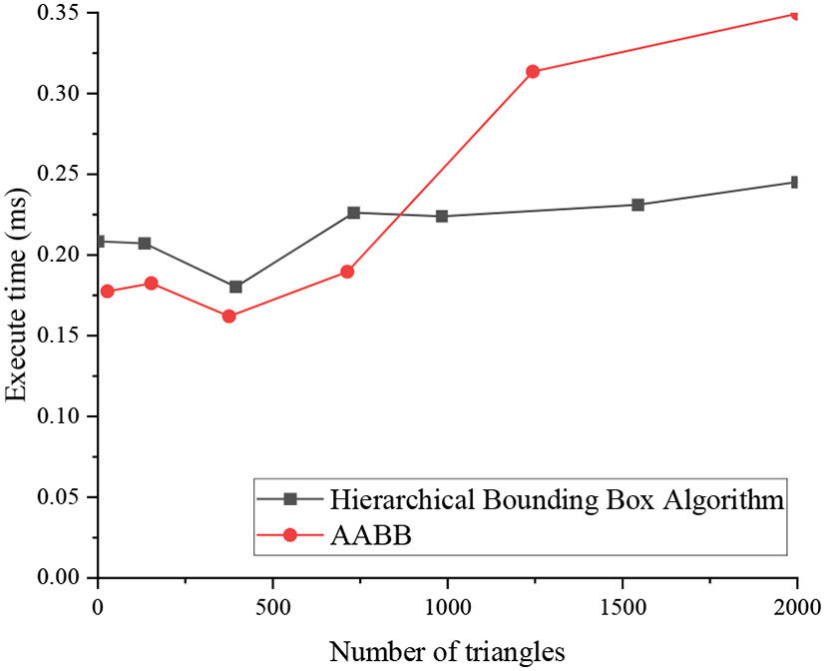
Time required for collision detection by two algorithms with different numbers of triangles.

The simulation experiment of the collision detection in the VR environment starts with data import, and this experiment uses the 3D building information read from the 3ds file, which is input to the cache of scene rendering. Then the corresponding bounding box model is obtained through the hierarchical bounding box algorithm, and OpenGL reads the data in the previous cache, initializes the scene, and displays the 3D model. At last, real-time collision detection is performed and the result is returned. Figure 9 denotes the time required for collision detection of the two algorithms in the object scene when the building model is composed of different numbers of triangles.

According to the results of the collision experiment in Figure 9, the adopted bounding box storage structure can reduce the storage requirements, and can also make the value of the bounding box of the internal node refer to the bounding box value of the root node, which requires additional consumption of certain retrieval time. Therefore, when the number of model triangles is small, the execution time of the applied hierarchical bounding box algorithm and Axially Aligned Bounding Box (AABB) algorithm is not much different. However, when the number of triangles corresponding to the model increases, the superiority of the hierarchical bounding box algorithm is obvious, which can achieve a balance between storage space and execution efficiency. Compared with the AABB algorithm, it is more economical in execution time, which is more beneficial to the HCI applied here.

### Analysis of test results of image performance

**Figure 10:**
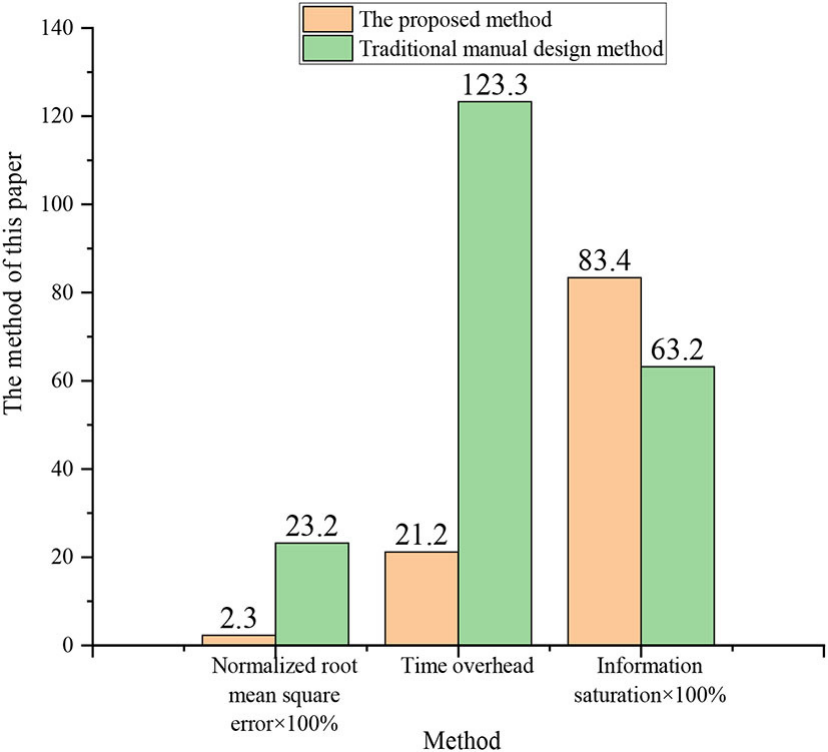
Comparison of test designs.

According to the results of the optimization of interior design and color information fusion in Figure 8B, the comparison of the normalized root mean square error (RMSE) of the design is carried out, and the time overhead and information saturation parameters are also tested, and the results are illustrated in Figure 10.

In Figure 10, compared with the software commonly used by designers in the comparison test, the RMSE and information saturation of the new 3D interior design are reduced by almost 0.2, and the time overhead is shortened to one-sixth of the original. It means that the more excellent the use of new 3D interior design, the stronger the ability to express design features, and the more comfortable the visual effects and user experience of the design are.

## Conclusion

Based on the requirements of entrepreneurship education for the interior environment, by using VR technology that benefits from the development of AI technology, a new type of 3D interior design scheme is proposed. VR technology can be effectively used to beautify the environment and optimize the combination of various modules, presenting different design effects, to meet the different needs of the interior environment of entrepreneurship education. It also adopts the feature information fusion method to implement the designed color image model, completes the edge contour detection and feature extraction, and then decomposes the image color pixel features. The 3dsMAX is utilized to realize the 3D modeling of the design, and modeling software is used for structural processing and scene interaction in UDK. The final comparison results reveal that compared with the traditional design method, the new distributed 3D design system has better visual effects, smaller error control, and better design cost savings.

Although the schematic diagram of the interior environment design of entrepreneurship education has been demonstrated, due to personal ability and time problems, the detection of edge contours and the data extraction of corresponding features are still relatively rough. Therefore, in the future, the design and development of the system should continue to be improved, and a more complete and practical solution should be designed to make a modest contribution to the indoor environment design of entrepreneurship education and beyond.

## Data availability statement

The raw data supporting the conclusions of this article will be made available by the authors, without undue reservation.

## Ethics statement

The studies involving human participants were reviewed and approved by Shandong University of Science and Technology Ethics Committee. The patients/participants provided their written informed consent to participate in this study. Written informed consent was obtained from the individual(s) for the publication of any potentially identifiable images or data included in this article.

## Author contributions

All authors listed have made a substantial, direct, and intellectual contribution to the work and approved it for publication.

## Funding

This work was supported by the funding of Star Project of Shandong University of Science and Technology's Education and Teaching Research in 2021: Research on Innovative Talents Training Model of Environmental Design Major Based on Interdisciplinary Under the Background of New Engineering (project number: QX2021M61).

## Conflict of interest

The authors declare that the research was conducted in the absence of any commercial or financial relationships that could be construed as a potential conflict of interest.

## Publisher's note

All claims expressed in this article are solely those of the authors and do not necessarily represent those of their affiliated organizations, or those of the publisher, the editors and the reviewers. Any product that may be evaluated in this article, or claim that may be made by its manufacturer, is not guaranteed or endorsed by the publisher.
